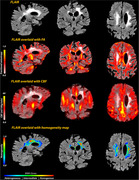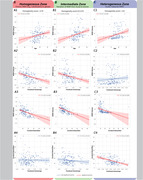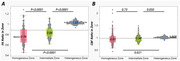# Characterizing White Matter Hyperintensity (WMH) Patterns with Texture‐Based Analysis

**DOI:** 10.1002/alz.093998

**Published:** 2025-01-09

**Authors:** Mohamad J. Alshikho, Clarissa Morales, Jeffrey D. Pyne, Erica Amarante, Patrick J. Lao, Indira C. Turney, Natalie C. Edwards, Yian Gu, Jennifer J. Manly, Richard Mayeux, Adam M. Brickman

**Affiliations:** ^1^ Taub Institute for Research on Alzheimer’s Disease and the Aging Brain, New York, NY USA; ^2^ Taub Institute for Research on Alzheimer’s Disease and the Aging Brain, Columbia University, New York, NY USA

## Abstract

**Background:**

White matter hyperintensities (WMHs) are areas of increased signal on T2‐weighted MRI scans. They vary in size, location, and intensity, suggesting different underlying conditions like small vessel disease and inflammation. This variation potentially links WMH to outcomes ranging from normal aging to severe neurological disorders. We used a texture analytic approach, Gray‐Level Co‐occurrence Matrix (GLCM), to characterize the intensity heterogeneity of WMHs. This method classifies WMHs into distinct zones based on homogeneity scores that represent uniformity in signal intensity. We explored associations among white matter microstructure, cerebral blood flow, and WMH volume within these zones.

**Method:**

One hundred sixty‐one participants from the Washington Heights‐Inwood Columbia Aging Project (WHICAP) were included. White matter hyperintensities were labeled using in‐house software, which also created normalized homogeneity maps through GLCM. These maps were used to define three WMH zones based on their degree of homogeneity (i.e., homogeneous, intermediate, and heterogeneous) (Figure 1). We examined the relationship among fractional anisotropy (FA), cerebral blood flow (CBF), and WMH volume within these WMH zones and compared CBF and FA values across the zones.

**Result:**

In homogeneous and intermediate WMH zones, there was no association between FA and CBF (rhomo. = 0.022, phomo. = 0.787; rinter. = ‐0.12, pinter. = 0.132). However, both FA and CBF negatively correlated with WMH volume in these zones (rFA‐homo. = ‐0.348, pFA‐homo.< 0.0001; rFA‐inter. = ‐0.538, pFA‐inter.< 0.0001; rCBF‐homo. = ‐0.36, pCBF‐homo.< 0.0001; rCBF‐inter. = ‐0.40, pCBF‐inter.< 0.0001). In the heterogeneous zone (Figure 2), FA and CBF were negatively correlated (rhetero. = ‐0.422, phetero.< 0.0001). CBF, but not FA, was associated with WMH volume (rCBF‐hetero. = ‐0.471, pCBF‐hetero.< 0.0001; rFA‐hetero. = 0.085, pFA‐hetero. = 0.300) In homogenous WMH regions, both [FARatio (0.776±0.009) and CBFRatio (0.96±0.014)] values were lower than in intermediate [FARatio (0.84±0.009) and CBFRatio (0.97±0.014)] and heterogeneous zones [FARatio: (1.029±0.009), CBFRatio: (1.002±0.014)].(Figure 3)

**Conclusion:**

Distinct correlation patterns exist between CBF and FA in WMH clusters defined by intensity heterogeneity. Homogeneous zones, likely reflect older lesions, show low microstructural integrity and minimal relationship with CBF, suggesting high gliosis. Heterogeneous zones, possibly reflecting recent white matter damage, show associations between CBF and microstructure, potentially indicating active inflammatory processes contributing to vascular injury.